# Intranasal dexmedetomidine versus midazolam for pediatric dental sedation: a pooled analysis of clinical trials

**DOI:** 10.3389/fmed.2026.1882122

**Published:** 2026-06-29

**Authors:** Jingyan Lyu, Cong Yu, Sisi Li, Jie Zeng

**Affiliations:** 1 The First Clinical College, Chongqing Medical University, Chongqing, China; 2 The Stomatological Hospital of Chongqing Medical University, Chongqing, China

**Keywords:** intranasal dexmedetomidine, intranasal midazolam, intranasal sedation, meta-analysis, pediatric dentistry

## Abstract

**Objectives:**

The optimal intranasal sedative agent for pediatric dental procedures remains a subject of debate. We systematically reviewed and meta-analyzed head-to-head trials comparing intranasal dexmedetomidine, intranasal midazolam, oral midazolam-based regimens, and intranasal combination regimens for pediatric dental sedation.

**Methods:**

PubMed, Web of Science Core Collection, and the Cochrane Central Register of Controlled Trials were searched (2014–2025) in accordance with the PRISMA 2020 statement. The primary outcome was procedure success rate; secondary outcomes included onset time, sedation duration, satisfactory sedation rate, satisfactory behavior rate, and adverse events. Dichotomous outcomes were pooled as risk ratios (RR) and continuous outcomes as mean differences (MD), using a DerSimonian-Laird random-effects model; the certainty of evidence was rated using GRADE. All meta-analyses were performed in RevMan Web 5.4.1, with single-arm rates presented descriptively.

**Results:**

Seventeen studies involving 1,215 children aged 1–14 years met the inclusion criteria, including 15 studies randomized controlled trials (RCTs) and 2 non-randomized studies. For the primary comparison of intranasal dexmedetomidine versus intranasal midazolam, the procedure success rate showed RR of 1.29 (95% CI: 0.86, 1.94; I^2^ = 68%; *p* = 0.22); onset time MD of 8.79 min (95% CI: 6.70, 10.87; I^2^ = 83%; *p* < 0.00001); sedation duration MD of 20.32 min; satisfactory sedation rate RR of 1.04 (95% CI: 0.92, 1.16; I^2^ = 35%; *p* = 0.54); and satisfactory behavior rate RR of 1.21 (95% CI: 0.94, 1.56; *p* = 0.14). For intranasal versus oral sedation regimens, the procedure success rate showed an RR of 1.07 (95% CI: 0.99, 1.16; *p* = 0.09). For combined intranasal regimens versus single intranasal agents, the procedure success rate showed RR of 1.25 (95% CI: 0.73, 2.13; *p* = 0.42). Risk of bias was low to moderate in most domains, with allocation concealment and blinding being the principal sources of unclear or high risk.

**Conclusion:**

Intranasal dexmedetomidine and intranasal midazolam are both clinically effective for pediatric dental sedation. Intranasal dexmedetomidine achieved a comparable procedure success rate and satisfactory sedation rate to intranasal midazolam, while demonstrating a slower onset and longer sedation duration. No statistically significant differences were detected in the procedure success rate between intranasal and oral sedation regimens or between combined and single intranasal regimens. Further adequately powered head-to-head RCTs with standardized outcome definitions are needed.

**Systematic review registration:**

https://osf.io/qc34e/.

## Introduction

Dental treatment can be a daunting experience for many children, often leading to significant anxiety, fear, and agitation (1). Dental fear/anxiety prevalence rates among children and adolescents range from 13.3 to 29.3% (2). Consequently, pediatric non-cooperation during dental procedures presents a pervasive challenge that significantly affects dentists’ ability in delivering treatment effectively. Pediatric sedation can minimize anxiety and pain and create better conditions for dental procedures. However, invasive sedation methods, such as intravenous cannulation, may itself cause considerable discomfort and anxiety therefore, needle-free pharmacological sedation is often more suitable for pediatric dental treatment. The non-invasive routes of administration, such as oral, buccal, and intranasal delivery, are therefore valuable alternatives. Among these alternative routes, nasal drug delivery offers several advantages: high bioavailability, a non-invasive, simple technique, bypassing the blood–brain barrier, avoiding hepatic first-pass metabolism, and good acceptance by children. The nasal mucosa, with its large surface area and rich vascularization, allows rapid and efficient drug absorption (3). Intranasal sedation administered by spray or drops can relieve children’s anxiety and fear during dental procedures (4, 5).

The intranasal sedatives in common use are primarily midazolam and dexmedetomidine (6, 7). Although several studies have evaluated the efficacy of intranasal sedation, evidence comparing the effectiveness and safety of different intranasal agents and of intranasal versus oral administration for dental procedures remains insufficient (8). As a premedication, intranasal dexmedetomidine has been associated with a lower incidence of perioperative respiratory adverse events and was considered more clinically appropriate than intranasal midazolam for sedation in children undergoing tonsillectomy and adenoidectomy (9). One systematic review and meta-analysis reported that intranasal dexmedetomidine is associated with a lower incidence of pediatric emergence delirium and better sedation levels than intranasal midazolam (10). Conversely, another meta-analysis suggested that intranasal midazolam has a faster onset and achieves sedation more rapidly than intranasal dexmedetomidine in pediatric dental sedation (11). A further systematic review and meta-analysis reported that intranasal midazolam, particularly when combined with ketamine or dexmedetomidine, provides a rapid onset and effective sedation for pediatric dental procedures (8). Moreover, current systematic reviews have not fully incorporated the most recent literature.

Thus, building on the existing pairwise meta-analyses by Swaminathan et al. (8) and Barot et al. (11), the present systematic review and meta-analysis aimed to evaluate the comparative effectiveness and safety of intranasal dexmedetomidine, intranasal midazolam, oral midazolam-based regimens, and intranasal combination regimens for mild-to-moderate sedation in children undergoing dental procedures, with an emphasis placed on head-to-head trial evidence published between January 2014 and December 2025.

## Materials and methods

### Protocol and registration

This systematic review and meta-analysis adhered to the Preferred Reporting Items for Systematic Reviews and Meta-Analyses (PRISMA) 2020 statement (12). A completed PRISMA 2020 checklist is provided in Supplementary Table S2. The analysis was not pre-registered in PROSPERO; the protocol supporting the head-to-head pairwise design has been deposited at the Open Science Framework (10.17605/OSF.IO/qc34e). All statistical synthesis and data analysis were conducted with Review Manager Web (RevMan Web, version 5.4.1, The Cochrane Collaboration).

### Eligibility criteria

Studies published between January 2014 and December 2025 were selected for this review in accordance with the PICOS framework and prespecified inclusion and exclusion criteria.

#### Inclusion criteria

- Population (P): Children (<18 years old), with or without intellectual disabilities, classified as ASA I or II by the American Society of Anesthesiologists, indicating good health or mild systemic disease, who required dental treatment and exhibited non-cooperation, anxiety, or severe phobia during dental care.
- Interventions (I): Studies focused on intranasal sedation with the administration of single or combined sedative agents via an intranasal route of sedative drug delivery for dental treatment.
- Comparison (C): Studies focused on placebo administration, oral sedation regimens using sedative agents, or an alternative intranasal sedation regimen.
- Outcomes (O): Primary and secondary outcomes included onset time, time to recovery, satisfactory sedation rate, satisfactory behavior rate, or procedure success rate.
·Studies approved by the ethics committee and reporting informed consent obtained from participants’ guardians.

#### Exclusion criteria

The exclusion criteria were as follows:

- Studies involving general anesthesia.
- Studies unrelated to intranasal sedation or those that did not report the outcomes of interest.
- Cross-over trials were not eligible for quantitative synthesis. In pediatric behavioral sedation, a child’s baseline anxiety and cooperation during the second treatment visit may be strongly influenced by the experience and outcome of the first sedation period, resulting in a carry-over (learning) effect that may not be eliminated by a wash-out interval. This violates the assumption of an independent, between-subject comparison on which pairwise pooling of parallel-group data depends, and restricting the analysis to first-period data alone would have discarded most of the reported information. Cross-over studies were therefore excluded from the meta-analysis but are listed in Supplementary Table S3 and discussed qualitatively in the Discussion, as they still provide clinically relevant evidence.
- Studies with only a design plan but no reported results.
- Studies written in languages other than English, case studies, reviews, and studies without a full text available.

### Literature search strategy

We performed a systematic search in PubMed, the Web of Science Core Collection, and Cochrane Central Register of Controlled Trials between January 2014 and December 2025.

The comprehensive search was conducted using advanced search filters:

- Keywords: (pediatric OR children) AND (intranasal) AND (sedative OR sedation) AND (dentistry OR dental).
- Time frame: January 2014 to December 2025.
- Total articles retrieved: 256.

Relevant studies were also found by searching the reference lists of included literature. Full reproducible search strings for each database are reported in Supplementary Table S1.

### Study selection

**Figure 1:**
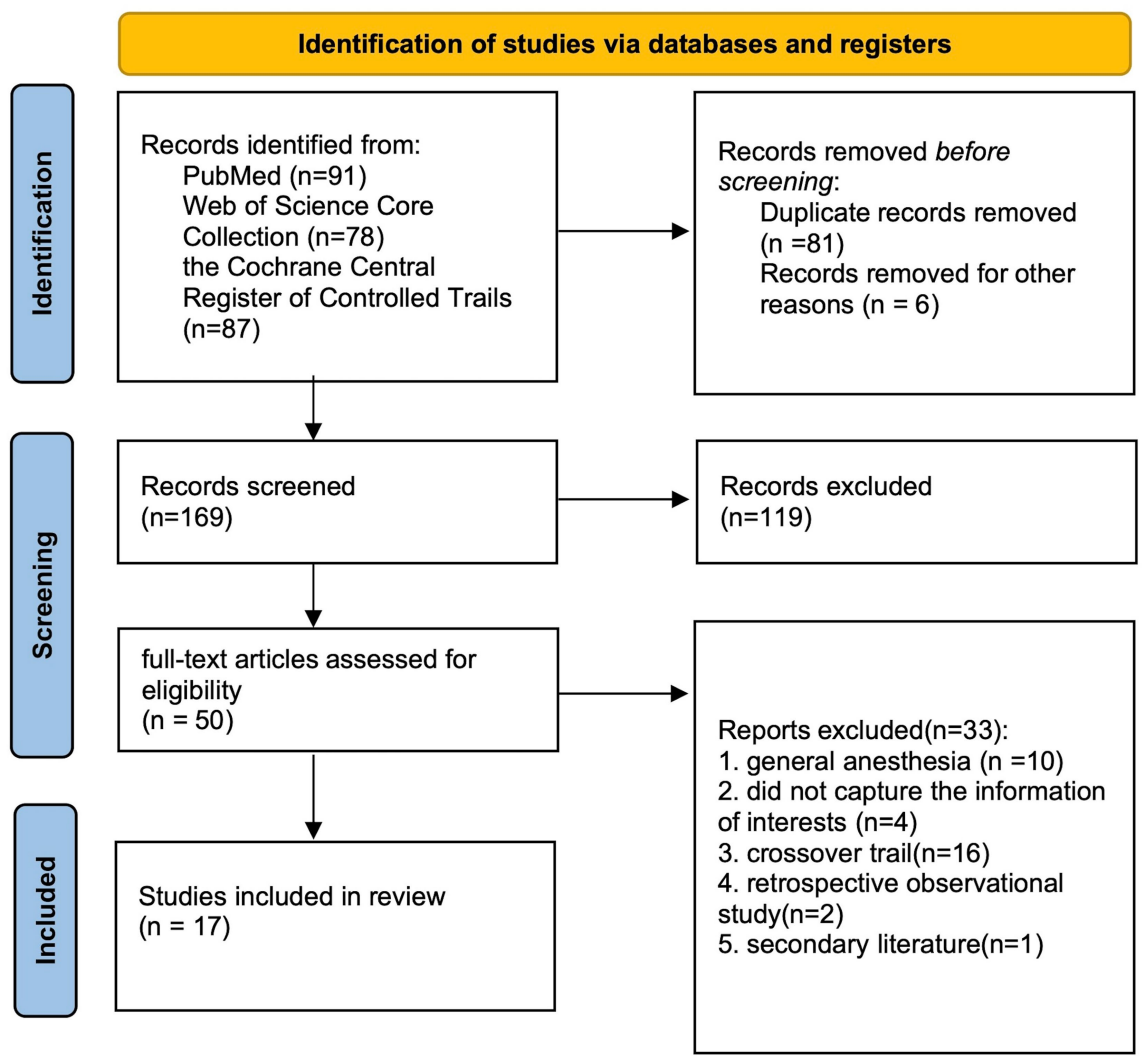
PRISMA flow diagram of the study selection process.

PubMed, Web of Science Core Collection, and the Cochrane Central Register of Controlled Trials were independently searched by two reviewers. The results were compared, and the search strategy was refined through consensus. All retrieved records were imported into EndNote and automatically deduplicated. The remaining titles and abstracts were independently screened by the same two reviewers, with disagreements resolved through discussion. When titles or abstracts provided insufficient information, full texts were obtained and assessed for eligibility. Every step of study selection followed the guidance in the Cochrane Handbook for Systematic Reviews of Interventions (13). The entire process, including the number of records identified, screened, and excluded, as well as the reasons for exclusion, is summarized in the PRISMA 2020 flow diagram (12) (Figure 1).

### Data extraction and outcomes

Two investigators independently extracted relevant data from all eligible studies using a pre-established extraction form. Any disagreements were resolved through group discussion until a consensus was achieved. The characteristics extracted and analyzed from studies that met the inclusion criteria are as follows: author, year of publication, study design, sample size and age range, intranasal sedative routes, onset time, sedation duration, satisfactory sedation rate, satisfactory behavior rate, and success rate.

Among these, the primary outcome was the success rate of performing dental treatment. Secondary outcomes were the onset time, defined as the time from administration of the sedative(s) to the achievement of adequate sedation; sedation duration, defined as the time from the achievement of adequate sedation to the time the patient was fully awake and alert; satisfactory sedation rate; satisfactory behavior rate; and adverse events. Sedation success rate, satisfactory sedation rate, and satisfactory behavior rate were expressed as percentages (%). Onset time and sedation duration were expressed as mean differences.

Notably, since the included studies adopted different scales to evaluate sedation status and patient behavior, we standardized these indicators for further pooled analysis.

Across the reviewed literature, sedation was classified using a variety of validated scales—the Wilson Sedation Scale (14), the University of Michigan Sedation Scale (UMSS) (15), and the Modified Observer’s Assessment of Alertness/Sedation (MOAA/S) (16), among others. Despite heterogeneous terminology, all instruments can be harmonized into five clinically meaningful sedation levels. Hence, sedation satisfaction was defined as the achievement of the following criteria:

- Scores ≥3 on the Wilson Sedation Scale.
- Scores ≥1 on the University of Michigan Sedation Scale (UMSS).
- Scores ≤4 on the Modified Observer’s Assessment of Alertness/Sedation (MOAA/S).

Behavioral outcomes were evaluated using a set of validated scales, including the OSUBRS (17), the Houpt Scale (18), the Frankl Behavior Rating Scale (18), and the Venham Anxiety/Behavior Scale (19). For this review, satisfactory behavior during treatment was defined as follows:

- Scores ≤2 on the OSUBRS.
- Scores ≥4 on the Houpt Scale.
- Scores ≥3 on the Frankl Behavior Rating Scale.
- Scores ≤1 on the Venham Anxiety/Behavior Scale.

### Risk-of-bias assessment of the included studies

**Figure 2:**
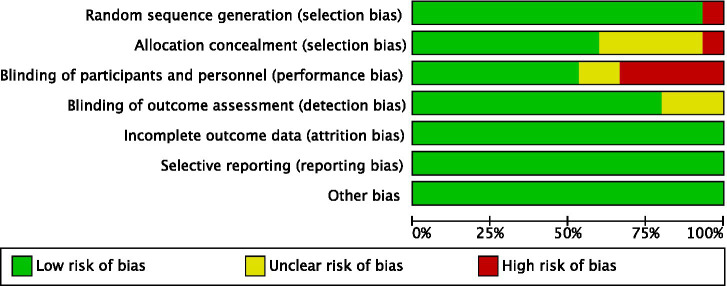
Risk-of-bias graph presented as a percentage of all included RCTs.

**Figure 3:**
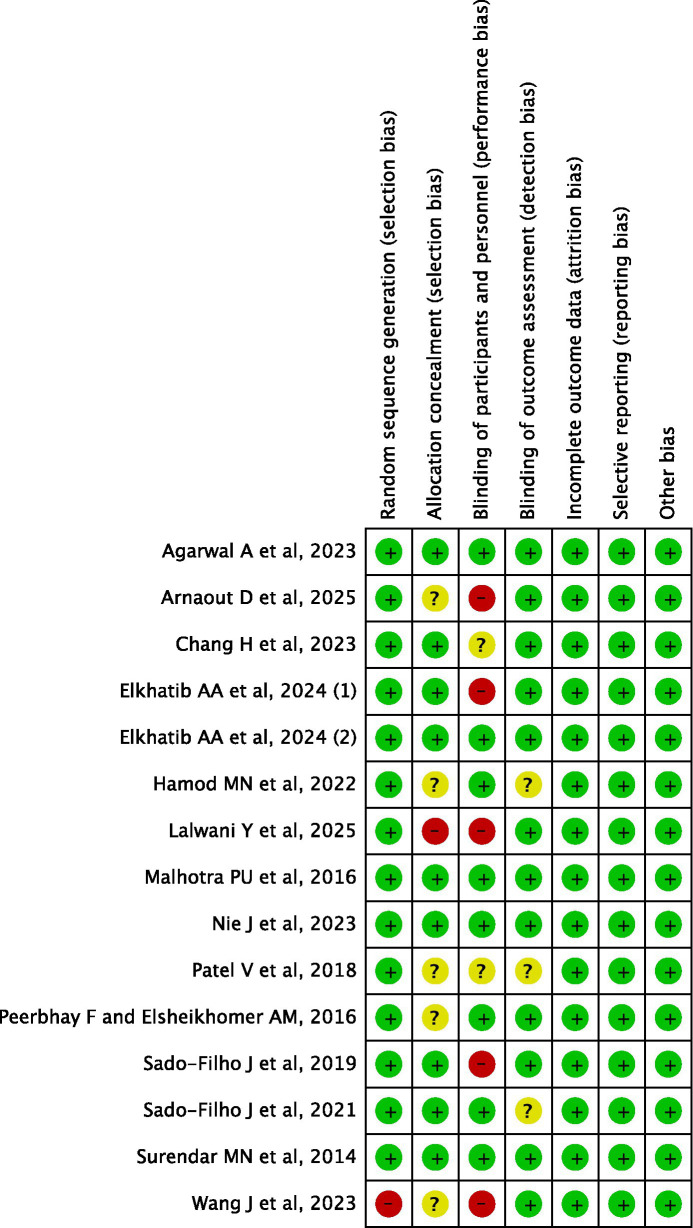
Risk-of-bias summary of all included RCTs.

The risk of bias was assessed independently by two reviewers. Among the 17 included studies, 15 were randomized controlled trials (RCTs) and 2 were non-randomized studies. RCTs were appraised with the Cochrane Collaboration’s risk-of-bias tool (20) across seven domains: random sequence generation, allocation concealment, blinding of participants and personnel, blinding of outcome assessment, incomplete outcome data, selective reporting, and other bias; each domain was rated as low, unclear, or high risk, and an overall judgment was assigned per study. The two non-randomized studies (21, 22) were appraised with the ROBINS-I tool across seven domains—confounding, selection, classification of interventions, deviations, missing data, outcome measurement, and selection of the reported result. Discrepancies were resolved by discussion or, when unresolved, by consultation with a third reviewer. The risk-of-bias results are presented graphically (Figure 2) and per-study (Figure 3).

### Statistical analysis

The included studies were grouped *a priori* by sedation regimen, with the primary analyses being head-to-head pairwise comparisons of (i) intranasal dexmedetomidine versus intranasal midazolam, (ii) intranasal dexmedetomidine or intranasal midazolam versus oral midazolam-based regimens, and (iii) intranasal combination regimens versus single intranasal agents. Single-arm pooled rates for each regimen were retained only as supplementary descriptive data and are not used for between-group inference.

For each pairwise comparison, dichotomous outcomes (procedure success rate, satisfactory sedation rate, satisfactory behavior rate, and adverse-event incidence) were pooled as risk ratios (RR) and continuous outcomes (onset time and sedation duration) as mean differences (MD), each with their 95% confidence intervals (CI). All pooled estimates were obtained with a DerSimonian–Laird random-effects model (23), irrespective of the observed I^2^, because the included trials varied substantially in patient population, drug dose, and procedure type. Heterogeneity was quantified by the chi-square test, I^2^ statistic (24), and the between-study variance τ^2^. For outcomes from a single trial, the study-level effect estimates and 95% CI from that trial are reported without further pooling and interpreted descriptively rather than as meta-analytic estimates. Single-arm proportions for each regimen were not pooled as a meta-analytic point estimate; per-study rates are summarized descriptively in Tables 1–3. All meta-analyses were performed using Review Manager Web (RevMan Web, version 5.4.1, The Cochrane Collaboration).

**Table 1:** Characteristics of included studies.

Study no.	Author, Year of publication	Design of the study	Sample size	Age, Years	Sedatives
1	Surendar MN et al., 2014 (27)	Randomized triple-blind controlled trial	84	4–14	Midazolam (intranasal) Dexmedetomidine (intranasal) Ketamine (intranasal)
2	Hitt JM et al., 2014 (22)	Non-comparative trial	20	3–7	Dexmedetomidine-sufentanil (intranasal)
3	Malhotra PU et al., 2016 (34)	Randomized doubled-bind controlled trial	42	3–9	Dexmedetomidine (intranasal) Midazolam-ketamine (oral) Saline (intranasal)–mango juice (oral)
4	Peerbhay F and Elsheikhomer AM, 2016 (28)	Randomized triple-blinded controlled trial	118	4–6	Midazolam (intranasal)
5	Patel V et al., 2018 (35)	Randomized controlled trial	44	4–9	Dexmedetomidine (intranasal) Dexmedetomidine (oral)
6	Sado-Filho J et al., 2019 (37)	Randomized triple-blind controlled trial	84	<7	Midazolam-ketamine (intranasal) Midazolam-Ketamine (oral) midazolam (oral)
7	Sado-Filho J et al., 2021 (36)	Randomized triple-blind controlled trial	88	1–7	Dexmedetomidine (intranasal) Dexmedetomidine-ketamine (intranasal)
8	Xin N et al., 2021 (21)	Single-center cohort study	150	3–10	Dexmedetomidine (intranasal) Esketamine (intranasal)
9	Hamod MN et al., 2022 (29)	Randomized controlled trial	20	5–11	Midazolam (intranasal) Dexmedetomidine (intranasal)
10	Chang H et al., 2023 (30)	Randomized controlled trial	66	3–9	Midazolam (intranasal) Midazolam (intramuscular)
11	Wang J et al., 2023 (40)	Randomized blinded controlled trial	60	2–6	Esketamine (intranasal)-Midazolam (oral)
12	Nie J et al., 2023 (38)	Randomized double-blinded controlled trial	83	3–12	Dexmedetomidine (intranasal)-Midazolam (oral) Midazolam (oral)
13	Agarwal A et al., 2023 (39)	Randomized triple-blinded controlled trial	128	4–9	Midazolam-ketamine (intranasal) Midazolam-fentanyl (intranasal) Dexmedetomidine-ketamine (intranasal) Dexmedetomidine-fentanyl (intranasal)
14	Elkhatib AA et al., 2024 (1) (31)	Randomized controlled trial	68	3–5	Midazolam (nebulized) (Atomized intranasal)
15	ElKhatib AA et al., 2024 (2) (31)	Randomized triple-blinded controlled trial	72	4–6	Midazolam (nebulized) Dexmedetomidine (nebulized) Dexmedetomidine-midazolam (nebulized)
16	Arnaout D et al., 2025 (32)	Randomized single-blinded trial	40	3–6	Midazolam (intranasal) Midazolam (buccal)
17	Lalwani Y et al., 2025 (33)	Randomized double-blinded trial	48	5–8	Midazolam (intranasal) Dexmedetomidine (intranasal)

**Table 2:** Efficacy outcomes of the included studies.

Study no.	Author, Year of publication	Subgroup	Sedative drugs	Dose	Onset time (min)	Sedation duration (min)	Satisfactory sedation rate	Satisfactory behavior rate	The success rate
1	Surendar MN et al., 2014 (27)	Surendar MN et al., 2014(a) (27)	Intranasal midazolam	0.2 mg/kg	10.43 ± 1.83	40.71 ± 2.45	71.4%	71.4%	61.9%
		Surendar MN et al., 2014(b) (27)	Intranasal ketamine	5 mg/kg	11.57 ± 2.18	44.19 ± 5.24	76.2%	76.2%	66.7%
		Surendar MN et al., 2014(c) (27)	Intranasal dexmedetomidine	1 μg/kg	18.24 ± 2.00	59.81 ± 5.89	90.5%	85.7%	81%
		Surendar MN et al., 2014(d) (27)	Intranasal dexmedetomidine	1.5 μg/kg	18.10 ± 2.00	62.24 ± 7.17	95.2%	90.5%	85.7%
2	Hitt JM et al., 2014 (22)	Hitt JM et al., 2014 (22)	Intranasal dexmedetomidine sufentanil	Dexmedetomidine:2 μg/kg (maximum 40 μg) Sufentanil:1 μg/kg (maximum 20 μg)	–	55 ± 21	–	–	–
3	Malhotra PU et al., 2016 (34)	Malhotra PU et al., 2016 (1) (34)	Intranasal dexmedetomidine	1 μg/kg	–	–	–	61.6%	–
		Malhotra PU et al., 2016 (2) (34)	Oral midazolam-ketamine	Midazolam:0.5 mg/kg Ketamine:5 mg/kg	–	–	–	83.3%	–
		Malhotra PU et al., 2016 (3) (34)	Control	Saline: 0.4 mL	–	–	–	0	–
4	Peerbhay F and Elsheikhomer AM, 2016 (28)	Peerbhay F and Elsheikhomer AM, 2016(a) (28)	Intranasal midazolam	0.3 mg/kg	35.21	16.5	–	–	–
		Peerbhay F and Elsheikhomer AM, 2016(b) (28)	Intranasal midazolam	0.5 mg/kg	36.45	18.8	–	–	–
5	Patel V et al., 2018 (35)	Patel V et al., 2018(a) (35)	Intranasal dexmedetomidine	2 μg/kg	8.82 ± 0.98	97.45 ± 7.03	27.27%	63.63%	–
		Patel V et al., 2018(b) (35)	Intranasal dexmedetomidine	2.5 μg/kg	7.00 ± 1.18	142.64 ± 45.63	81.81%	90.90%	–
		Patel V et al., 2018(c) (35)	Oral dexmedetomidine	4 μg/kg	47.36 ± 23.43	87.18 ± 43.20	0	18.2%	–
		Patel V et al., 2018(d) (35)	Oral dexmedetomidine	5 μg/kg	34.45 ± 27.37	75.36 ± 59.95	0	27.3%	–
6	Sado-Filho J et al., 2019 (37)	Sado-Filho J et al., 2019(a) (37)	Intranasal midazolam-ketamine	Midazolam: 0.2 mg/kg (maximum 5.0 mg) Ketamine: 4.0 mg/kg (maximum 100.0 mg)	–	–	–	50.0%	92.9%
		Sado-Filho J et al., 2019(b) (37)	Oral midazolam-ketamine	Ketamine: 4.0 mg/kg (maximum 100.0 mg) Midazolam: 0.5 mg/kg (maximum 5.0 mg)	–	–	–	46.4%	89.3%
		Sado-Filho J et al., 2019(c) (37)	Oral midazolam	1.0 mg/kg (maximum 20.0 mg)	–	–	–	32.1%	85.7%
7	Sado-Filho J et al., 2021 (36)	Sado-Filho J et al., 2021(a) (36)	Intranasal dexmedetomidine	2.5 μg/kg (maximum 100 μg)	45.0 ± 8.15	36.5 ± 11.48	97.8%	55.2%	90.9%
		Sado-Filho J et al., 2021(b) (36)	Intranasal dexmedetomidine-ketamine (20 min)	Dexmedetomidine: 2 μg/kg (maximum 100 μg) Ketamine: 1 μg/kg (maximum 100 mg)	44.0 ± 7.19	62.5 ± 27.04	90.9%	58.4%	90.9%
8	Xin N et al., 2021 (21)	Xin N et al., 2021(a) (21)	Intranasal esketamine	0.5 mg/kg	12.45 ± 2.27	60.25 ± 4.21	68.42%	63.16%	*55.26%*
		Xin N et al., 2021(b) (21)	Intranasal dexmedetomidine	1 μg/kg	19.45 ± 2.41	58.45 ± 4.66	64.86%	62.16%	56.76%
		Xin N et al., 2021(c) (21)	Intranasal dexmedetomidine	1.5 μg/kg	19.32 ± 2.36	61.45 ± 5.71	78.05%	75.61%	75.61%
		Xin N et al., 2021(d) (21)	Intranasal dexmedetomidine	2.0 μg/kg	17.56 ± 2.14	63.14 ± 4.58	79.41%	82.35%	73.53%
9	Hamod MN et al., 2022 (29)	Hamod MN et al., 2022(a) (29)	Intranasal midazolam	0.2 mg/kg	–	–	100%	70%	100%
		Hamod MN et al., 2022(b) (29)	Intranasal dexmedetomidine	1 μg/kg	–	–	100%	80%	100%
10	Chang H et al., 2023 (30)	Chang H et al., 2023(a) (30)	Intranasal midazolam (IN)	0.3 mg/kg	13.33 ± 2.74	13.48 ± 3.67	–	–	
		Chang H et al., 2023(b) (30)	Intramuscular midazolam (IM)	0.3 mg/kg	13.39 ± 3.17	11.70 ± 4.57	–	–	
11	Wang J et al., 2023 (40)	Wang J et al., 2023 (40)	Intranasal esketamine with oral midazolam	Midazolam: 0.5 mg/kg Esketamine: 1.99 mg/kg	43.7 ± 6.9	–	–	–	88.33%
12	Nie J et al., 2023 (38)	Nie J et al., 2023(a) (38)	Intranasal dexmedetomidine with oral Midazolam	dexmedetomidine:2 | 17.5 ± 2.4 | − | 77.5% | − | 100% μg/kg | | | | | | | | | | (maximum 100 μg) | | | | | | | | | | | midazolam 0.5 mg/kg | | | | | | | | | | (maximum 20.0 mg) | | | | |					
		Nie J et al., 2023(b) (38)	oral midazolam	0.5 mg/kg (maximum 20.0 mg)	15.7 ± 1.8	–	48.8%	–	93.0%
13	Agarwal A et al., 2023 (39)	Agarwal A et al., 2023(a) (39)	Intranasal midazolam-ketamine	Midazolam: 0.2 mg/kg (maximum 5.0 mg) Ketamine: 4.0 mg/kg (maximum 100 mg)	9.60 ± 1.65	45.71 ± 5.54	75.0%	81.25%	78.1%
		Agarwal A et al., 2023(b) (39)	Intranasal dexmedetomidine-ketamine	Dexmedetomidine: 1 μg/kg (maximum 100 μg) Ketamine: 1 mg/kg (maximum 100 mg)	17.10 ± 2.18	80.36 ± 5.71	100%	93.75%	93.8%
		Agarwal A et al., 2023(c) (39)	Intranasal midazolam-fentanyl	Midazolam: 0.2 mg/kg (maximum 10 mg) Fentanyl: 2 μg/kg (maximum 100 μg)	10.79 ± 1.53	40.19 ± 4.93	71.9%	78.13%	59.4%
		Agarwal A et al., 2023(d) (39)	Intranasal dexmedetomidine-fentanyl	Dexmedetomidine: 1 μg/kg (maximum 100 μg) Fentanyl: 1.5 μg/kg (maximum 100 μg)	18.24 ± 2.07	70.43 ± 6.19	93.7%	93.75%	84.4%
14	Elkhatib AA et al., 2024 (1) (31)	Elkhatib AA et al., 2024 (1)(a) (31)	Nebulized midazolam	0.5 mg/kg	–	–	100%	58.8%	100%
		Elkhatib AA et al., 2024 (1)(b) (31)	Atomized intranasal midazolam	0.3 mg/kg	–	–	76.5%	38.2%	100%
15	ElKhatib AA et al., 2024 (2) (31)	ElKhatib AA et al., 2024 (2)(a) (31)	Nebulized dexmedetomidine	5 μg/kg	17.08 ± 5.88	–	100%	–	50.0%
		ElKhatib AA et al., 2024 (2)(b) (31)	Nebulized dexmedetomidine with nebulized midazolam	Dexmedetomidine: 3 μg/kg Midazolam: 0.3 mg/kg	8.33 ± 4.34	–	100%	–	58.3%
		El Khatib AA et al., 2024 (2)(c) (31)	Nebulized midazolam	0.5 mg/kg	11.88 ± 5.48	–	100%	–	16.7%
16	Arnaout D et al., 2025 (32)	Arnaout D et al., 2025(a) (32)	Intranasal midazolam	0.3 mg/kg	13.15	23.80	80%	65%	–
		Arnaout D et al., 2025(b) (32)	Buccal midazolam	0.3 mg/kg	13.73	22.90	85%	75%	–
17	Lalwani Y et al., 2025 (33)	Lalwani Y et al., 2025(a) (33)	Intranasal midazolam	0.25 mg/kg	30.79 ± 3.04	–	–	–	–
		Lalwani Y et al., 2025(b) (33)	Intranasal dexmedetomidine	1.5 μg/kg	41.42 ± 3.15	–	–	–	–

**Table 3:** Adverse events by sedation regimen.

Adverse events	Intranasal midazolam (*N* = 162)	Intranasal dexmedetomidine (*N* = 188)	Oral midazolam-based regimens (*N* = 218)	Intranasal combination regimens (*N* = 176)
Nausea-vomiting	0 (0.0%)	1 (0.5%)	28 (12.8%)	11 (6.3%)
Hypoxia	1 (0.6%)	0 (0.0%)	0 (0.0%)	1 (0.6%)
Drowsiness	0 (0.0%)	0 (0.0%)	3 (1.4%)	1 (0.6%)
Burning sensation	10 (6.2%)	0 (0.0%)	0 (0.0%)	0 (0.0%)
Total	11 (6.8%)	1 (0.5%)	31 (14.2%)	13 (7.5%)

Publication bias was assessed through visual inspection of funnel plots for any outcome with at least 10 contributing studies; because no outcome reached this threshold, formal funnel-plot evaluation and Egger’s regression test (25) were not performed, which is acknowledged as a limitation. A two-sided *p*-value of <0.05 was considered statistically significant.

The certainty of evidence for each outcome was rated with the Grading of Recommendations Assessment, Development and Evaluation (GRADE) approach (26). Evidence derived from randomized trials was initially rated at high certainty and then rated down by one or more levels based on risk of bias, inconsistency, indirectness, imprecision, and publication bias; the resulting certainty was classified as high, moderate, low, or very low. Because all outcomes were informed by fewer than 10 trials, publication bias could not be formally assessed and was therefore not used as a separate reason for rating down. The GRADE judgments for all outcomes across the three comparisons are presented in the Summary of Findings table (Supplementary Table S4).

## Results

### Selection of studies

An electronic search of PubMed, Web of Science Core Collection, and Cochrane Central Register of Controlled Trials yielded 256 records. After removing 81 duplicate records and 6 irrelevant records for other reasons, a total of 169 records were retained for preliminary screening. Subsequently, 119 records were excluded following title and abstract screening. The remaining 50 full-text articles were assessed for eligibility, of which 33 were subsequently excluded for reasons including, but not limited to, general anesthesia (*n* = 10), failure to capture the information of interests (*n* = 4), crossover trial design (*n* = 16; listed in Supplementary Table S3), retrospective observational study (*n* = 2), or secondary literature (*n* = 1). Ultimately, 17 studies met all inclusion and exclusion criteria and were incorporated into the systematic review and meta-analysis. Details of study screening and selection are described in the PRISMA 2020 flow diagram (12) (Figure 1).

### Basic characteristics of enrolled studies

A total of 17 studies met the inclusion criteria and were incorporated in the present review. All studies were published between January 2014 and December 2025. Fifteen were parallel-group RCTs, nine of which were double- or triple-blinded; one study was a single-center cohort investigation, and one was a non-comparative clinical trial. The included studies enrolled 1,215 children aged 1–14 years. The included studies investigated various sedation protocols with different drugs, doses, and administration routes for different clinical procedures. Outcome measures included onset time, time to recovery, satisfactory sedation rate, satisfactory behavior rate, and the procedure success rate. Detailed study characteristics are summarized in Tables 1, 2.

### Risk-of-bias assessment

Risk of bias was assessed independently by two reviewers using the Cochrane Collaboration’s risk-of-bias tool for the 15 RCTs and ROBINS-I for the 2 non-randomized studies. The results across the seven domains are summarized graphically in Figure 2 and per study in Figure 3. A majority of RCTs were rated as low risk for random sequence generation, incomplete outcome data, selective reporting, and other bias, and blinding of outcome assessment was rated as low risk in a majority of trials, whereas allocation concealment and blinding of participants and personnel were the principal sources of unclear or high risk. The two non-randomized studies (21, 22) demonstrated a serious risk of bias in the confounding domain according to ROBINS-I because of non-randomized allocation and limited covariate adjustment; their data were retained in the descriptive summary but should be interpreted with caution given this serious risk of bias.

### Results of the individual studies and meta-analysis

#### Sedation drug doses

We identified four types of sedation drugs, including intranasal midazolam, intranasal dexmedetomidine, oral sedative, and intranasal sedation combined with other sedatives. A total of eight studies (318 patients; Tables 1, 2) reported the use of intranasal midazolam 0.2–0.5 mg/kg as a sedative technique for pediatric dental treatment (5, 27–33). Intranasal dexmedetomidine (1–5 μg/kg) was used in nine studies (290 patients; Tables 1, 2) (21, 27, 29, 31, 33–36). The use of oral sedatives for pediatric dental treatment is described in four studies (133 patients; Tables 1, 2) (34, 35, 37, 38). Midazolam (0.5–1 mg/kg), ketamine (4–5 mg/kg), and dexmedetomidine (4–5 μg/kg) were used as oral sedatives. Seven studies (387 patients; Tables 1, 2) described the use of combined sedatives for pediatric dental treatment. Combined intranasal sedation regimens included intranasal dexmedetomidine (2 μg/kg) with sufentanyl (1 μg/kg) (22), intranasal dexmedetomidine (1 μg/kg) with fentanyl (1.5 μg/kg) (39), intranasal midazolam (0.2 mg/kg) with fentanyl (2 μg/kg) (39), intranasal midazolam (0.2 mg/kg) with ketamine (4.0 mg/kg) (37, 39), intranasal dexmedetomidine (1–2 μg/kg) and ketamine (1 mg/kg) (36, 39), intranasal midazolam (0.3 mg/kg) with dexmedetomidine (3 μg/kg) (31), intranasal esketamine (1.99 mg/kg) with oral midazolam (0.5 mg/kg) (40), and intranasal dexmedetomidine (2 μg/kg) with oral midazolam (0.5 mg/kg) (38).

#### The primary outcome

**Figure 4:**
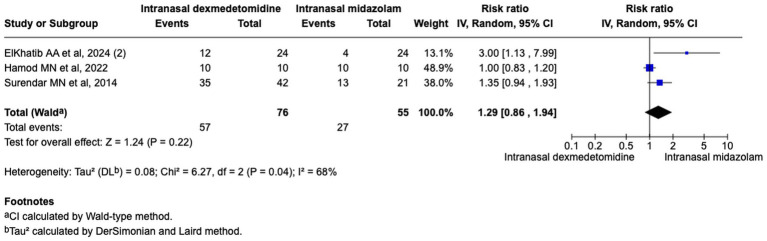
Pairwise meta-analysis of intranasal dexmedetomidine versus intranasal midazolam for procedure success rate.

In the comparison of intranasal dexmedetomidine versus intranasal midazolam, the procedure success rate in the intranasal midazolam group ranged from 16.67 to 100.0%. The pooled average success rate was 49.09%. The success rate of intranasal dexmedetomidine ranged from 50.0 to 100.0%, with a pooled average success rate of 75.00%. When comparing the two intranasal sedatives (k = 3, *n* = 131), the pooled risk ratio (RR) was 1.29 (95% CI: 0.86, 1.94; I^2^ = 68%; *p* = 0.22), with no statistically significant difference between groups; the wide confidence interval crossing 1 and the substantial heterogeneity indicate that this estimate should be interpreted with caution (Figure 4).

**Figure 5:**
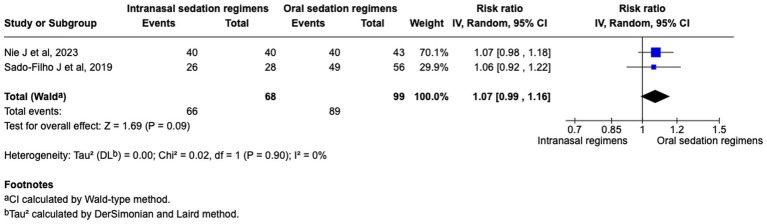
Pairwise meta-analysis of intranasal versus oral sedation regimens for procedure success rates.

In the analysis between intranasal and oral sedation, it was observed that the procedure success rate for oral regimens ranged from 87.50 to 93.02%, with a pooled average success rate of 91.0%. Meanwhile, the success rate of intranasal protocols ranged from 92.86 to 100.0%. When comparing intranasal sedation regimens with oral sedation regimens (k = 2, *n* = 167), the pooled RR was 1.07 (95% CI: 0.99, 1.16; I^2^ = 0%; *p* = 0.09), indicating no statistical difference (Figure 5).

**Figure 6:**
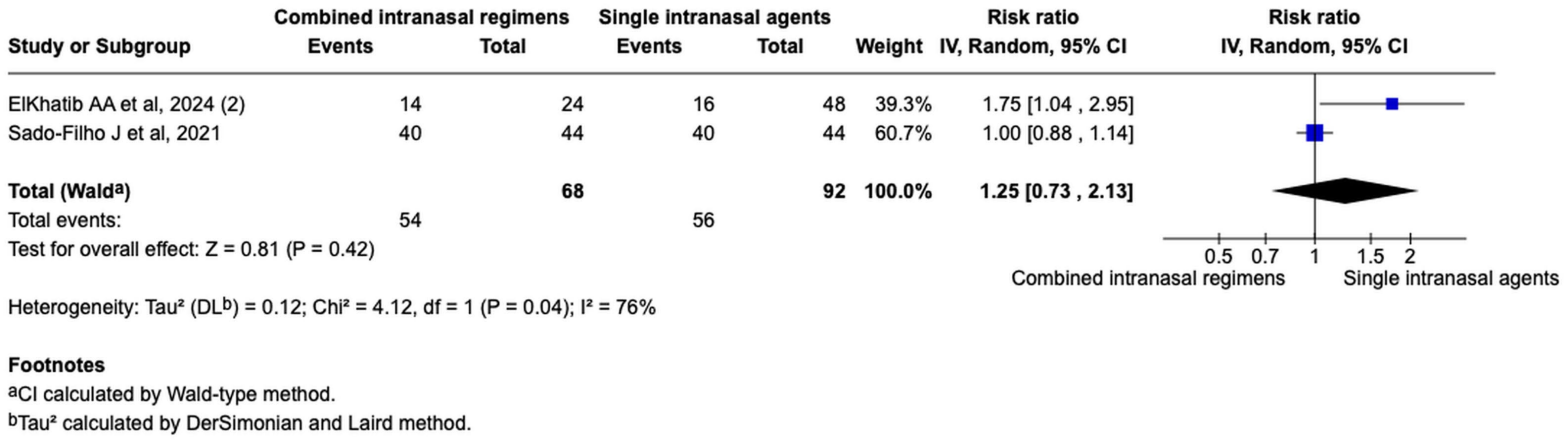
Pairwise meta-analysis of comparing intranasal regimens versus single intranasal agents for procedure success rate.

Regarding combined intranasal sedation regimens versus single-agent intranasal sedation, the procedure success rate of single-agent intranasal sedation ranged from 33.33 to 90.90%, while that of combined regimens ranged from 58.33 to 90.90%. The pooled average success rates were 60.87 and 79.41%, respectively. When comparing combined intranasal regimens with single intranasal agents (k = 2, *n* = 160), the pooled RR was 1.25 (95% CI: 0.73, 2.13; I^2^ = 76%; *p* = 0.42), showing a lack of statistical difference between the two groups; given the wide confidence interval and high heterogeneity, this comparison is regarded as hypothesis-generating only (Figure 6).

#### The secondary outcomes

##### The onset time

Sedation onset time was evaluated across three pairwise meta-analyses.

**Figure 7:**
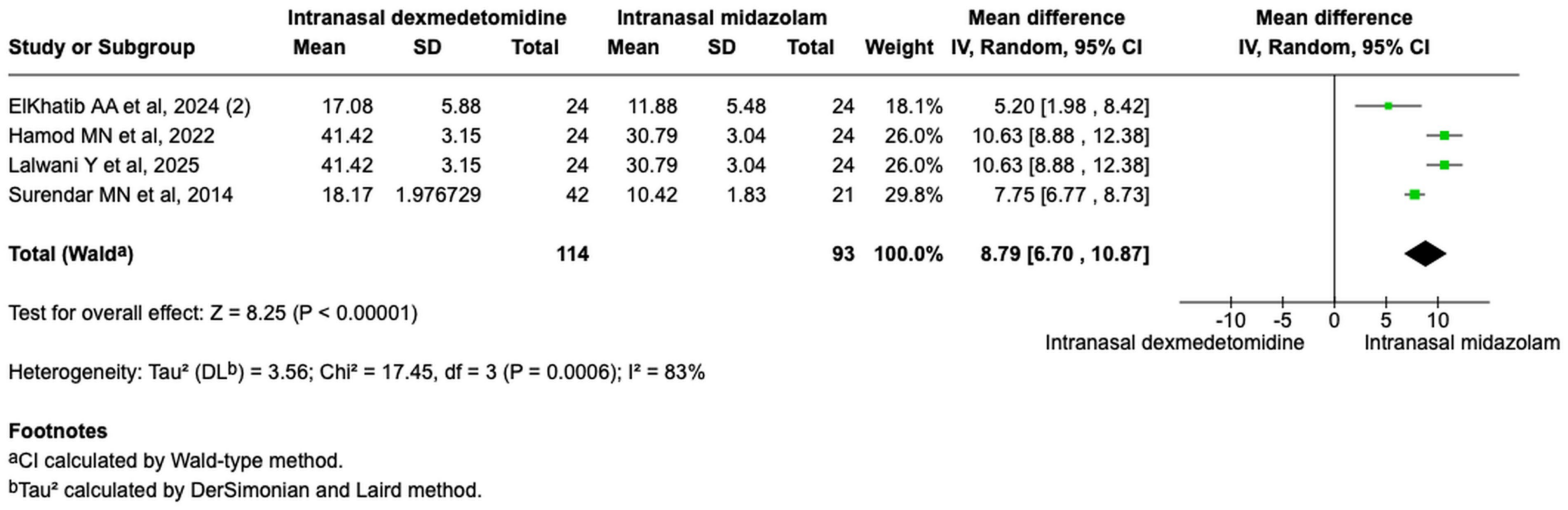
Pairwise meta-analysis comparing intranasal dexmedetomidine versus intranasal midazolam for onset time.

Four studies (total *n* = 207) comparing intranasal dexmedetomidine with intranasal midazolam yielded a pooled mean difference (MD) of 8.79 min (95% CI: 6.70, 10.87; I^2^ = 83%, *p* < 0.00001), revealing a statistically significant difference and indicating a longer sedation onset time in the dexmedetomidine group (Figure 7).

Two trials (total *n* = 127) comparing intranasal and oral sedation regimens reported a combined MD of −15.17 min (95% CI: −49.26, 18.92; I^2^ = 97%). No statistically significant difference was detected between the two groups.

Data from two relevant studies (*n* = 160) were pooled to compare combined and single intranasal regimens, yielding an MD of −3.68 min (95% CI: −8.72, 1.36; I^2^ = 84%). There was no statistically significant distinction between the two protocols.

##### The sedation duration

The outcome of sedation duration was examined in three pairwise comparisons.

**Figure 8:**
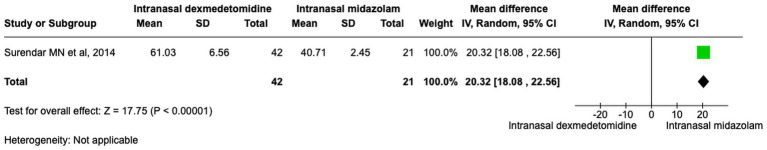
Pairwise meta-analysis comparing intranasal dexmedetomidine versus intranasal midazolam for sedation duration.

For the comparison between intranasal dexmedetomidine and intranasal midazolam, only one trial (*n* = 63) reported sedation duration, with a mean difference of 20.32 min (95% CI: 18.08, 22.56; *p* < 0.00001) in favor of intranasal dexmedetomidine; this single-trial estimate is descriptive and was not pooled (Figure 8).

The comparison of intranasal versus oral sedation regimens was based on a single trial (*n* = 44), which reported a mean difference of 38.78 min (95% CI: 11.74, 65.81; *p* = 0.005) in favor of intranasal sedation.

For combined versus single intranasal regimens, a single trial (*n* = 88) reported a sedation duration, with an MD of 26.00 min (95% CI: 17.32, 34.65; *p* < 0.00001), with combined regimens producing a longer sedation duration.

##### The satisfactory sedation rate

**Figure 9:**
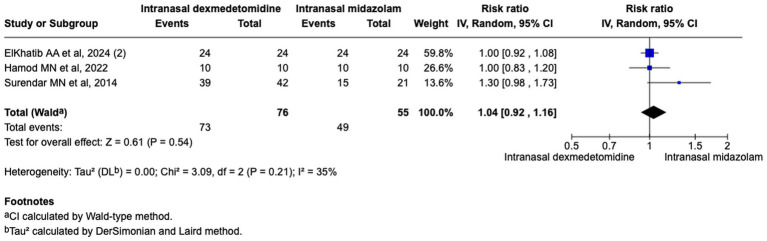
A pairwise meta-analysis comparing intranasal dexmedetomidine versus intranasal midazolam for satisfactory sedation rate.

The pairwise meta-analysis comparing intranasal dexmedetomidine with intranasal midazolam included three studies (*n* = 131). The pooled RR was 1.04 (95% CI: 0.92, 1.16; I^2^ = 35%; *p* = 0.54). No statistically significant difference was observed between the two groups. Across the included trials, the satisfactory sedation rate of intranasal midazolam ranged from 71.43 to 100.0%, whereas that of intranasal dexmedetomidine ranged from 92.86 to 100.0% (Figure 9).

The comparison between intranasal and oral sedation regimens comprised two studies (*n* = 127). The pooled RR was 4.41 (95% CI: 0.32, 60.04; I^2^ = 73%; *p* = 0.26). The very wide confidence interval crossing 1 indicates that no firm conclusion can be drawn despite the apparently large point estimate. The satisfactory sedation rate of oral sedation ranged from 0 to 48.88%, whereas the corresponding rate for intranasal sedation ranged from 54.54 to 77.50%.

The analysis of combined intranasal regimens versus single intranasal agents included two studies (*n* = 160). The pooled RR was 0.98 (95% CI: 0.91, 1.04; I^2^ = 27%; *p* = 0.48). There was no statistically significant difference between the two groups. Single intranasal agents achieved satisfactory sedation rates ranging from 97.73 to 100.00%, whereas combined sedation regimens ranged from 90.90 to 100.00%.

##### The satisfactory behavior rate

**Figure 10:**
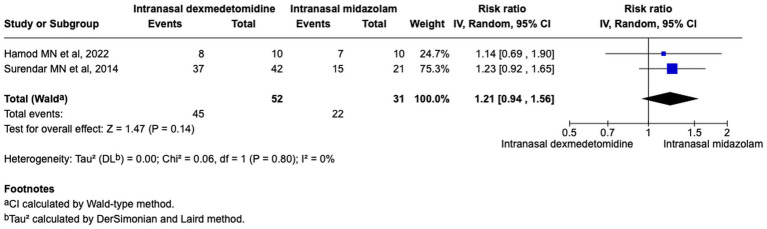
Pairwise meta-analysis comparing intranasal dexmedetomidine versus intranasal midazolam for satisfactory behavior rate.

A comparison between intranasal dexmedetomidine and intranasal midazolam was synthesized (k = 2, *n* = 83). The pooled RR was 1.21 (95% CI: 0.94, 1.56; I^2^ = 0%; *p* = 0.14). No statistically significant difference was observed between the groups. Across trials, satisfactory behavior rates for intranasal midazolam ranged from 70.00 to 71.43%, whereas those for intranasal dexmedetomidine ranged from 80.00 to 88.10% (Figure 10).

For intranasal versus oral sedation (k = 2, *n* = 128), the pooled RR was 1.97 (95% CI: 0.76, 5.14; I^2^ = 76%; *p* = 0.16). The difference between the two groups did not reach statistical significance, and the wide confidence interval crossing 1, together with the high heterogeneity, indicates that the apparent advantage of intranasal sedation cannot be regarded as established. Satisfactory behavior rates for oral sedation ranged between 22.72 and 39.29%, while intranasal sedation ranged from 50.00 to 77.27%.

For combined versus single intranasal sedation, a single trial (k = 1, *n* = 88) reported satisfactory behavior rates of 54.55% for single-agent regimens and 59.09% for combined regimens (RR 1.08, 95% CI: 0.75, 1.56; *p* = 0.67).

##### Adverse events

In our study, we evaluated several side effects, including nausea/vomiting, prolonged drowsiness, a burning sensation, hypoxia, and changes in several common hemodynamic parameters. Complications were rare and not clinically significant.

After the administration of intranasal midazolam 0.3 mg/kg, hypoxia was reported in 3.03% of children aged 3–9 years who underwent laceration repair of the maxillofacial region. Emergence delirium was also described in this cohort, and all events resolved with appropriate intervention. A burning sensation was reported in 9% of children receiving intranasal midazolam 0.3 or 0.5 mg/kg.

Among patients who received intranasal dexmedetomidine (2 μg/kg) combined with sufentanil (1 μg/kg), parents reported one child with prolonged drowsiness and one child who vomited at home (22). In 88 patients with intranasal dexmedetomidine alone or combined with ketamine, 18.2% participants had an adverse event, including 15.9% nausea and vomiting and 1.1% desaturation (36). All of them were minor.

The overall incidence of adverse events was 16.3% in participants who received 0.5 mg/kg oral midazolam and 20.0% in those who received 2 μg/kg intranasal dexmedetomidine combined with 0.5 mg/kg oral midazolam. All adverse event symptoms resolved after rest (38).

A descriptive summary of adverse events by sedation regimen across included studies is provided in Table 3. For intranasal versus oral sedation, pooled adverse-event incidence yielded RR of 0.93 (95% CI: 0.51, 1.72; I^2^ = 0%; *p* = 0.83; k = 2, *n* = 167). For combined versus single intranasal regimens, a single trial (k = 1, *n* = 88) reported RR of 1.29 (95% CI: 0.53, 3.15; *p* = 0.58).

### Certainty of evidence (GRADE)

The GRADE certainty of evidence is summarized in. All pooled estimates were derived from randomized controlled trials; the two non-randomized studies contributed only to the single-arm descriptive data. Across the three comparisons, certainty ranged from moderate to very low. Eight outcomes were graded as moderate, including most outcomes in the comparison between intranasal dexmedetomidine midazolam, which were rated down only for imprecision because their contributing trials were at low risk of bias in the relevant domains and showed little or no inconsistency. The remaining outcomes were rated down further for inconsistency (high I^2^) and/or risk of bias when the contributing trials were judged to have high or unclear risk on a relevant domain; the comparison of the satisfactory sedation rate of intranasal versus oral regimens (RR 4.41, 95% CI 0.32–60.04) and outcomes informed by a single small trial with a wide interval were graded as very low. No outcome reached high certainty because all estimates were derived from a small number of trials. These ratings indicate that the present estimates should be regarded as preliminary and that further evidence may change the magnitude and, for the lowest-certainty outcomes, the direction of effect.

## Discussion

This systematic review and meta-analysis synthesized a decade of pediatric dental sedation trials (2014–2025) involving 1,215 children aged 1–14 years. In contrast to the two recent pairwise meta-analyses by Swaminathan et al. (8) and Barot et al. (11), our primary analyses were head-to-head pairwise comparisons rather than single-arm pooled rates to avoid between-study confounding by case-mix, drug dose, and procedure type. The head-to-head results from pairwise meta-analyses were synthesized across three comparative groups: (i) intranasal dexmedetomidine versus intranasal midazolam – RR = 1.29 for procedure success, MD = 8.79 min for onset time, MD = 20.32 min for sedation duration, RR = 1.04 for satisfactory sedation, RR = 1.21 for satisfactory behavior; (ii) intranasal vs. oral sedation regimens – RR = 1.07 for procedure success rate, MD = −15.17 min for onset time, MD = 38.78 min for sedation duration, RR = 4.41 for satisfactory sedation, RR = 1.97 for satisfactory behavior, RR = 0.93 for adverse events; (iii) combined intranasal regimens vs. single intranasal agents – RR = 1.25 for procedure success rate, MD = −3.68 min for onset time, MD = 26.00 min for sedation duration, RR = 0.98 for satisfactory sedation, RR = 1.08 for satisfactory behavior, RR = 1.29 for adverse events. Single-arm summary rates per regimen are described qualitatively in Tables 1, 2 but were not pooled because contributing studies differ systematically in patient population, dose, and procedure type. The clinical implications of each regimen are discussed below.

### Intranasal midazolam

Intranasal midazolam has been used in pediatric sedation for more than 30 years (41). In this meta-analysis, intranasal midazolam 0.2–0.5 mg/kg during pediatric dental procedures was associated with an average success rate of 58%. The onset time ranged from 10.43 to 36.45 min, and the duration from 23.8 to 40.7 min. The average satisfactory sedation rate was 76%. The average satisfactory behavior rate was 59%. Intranasal midazolam (0.1–0.2 mg/kg) was also used as a pediatric premedication for general anesthesia (9), acute seizures (42), and emergence delirium (10). Intranasal midazolam (0.2 mg/kg) for the home treatment of acute seizures in children achieved a satisfaction score of 9.3 on an 11-point nominal scale (0, not satisfied, and 10, greatly satisfied) (42). A comparison of nasal and oral midazolam sedation in pediatric dental patients showed that the onset of sedation was much shorter with the intranasal route, whereas the mean duration of sedation was significantly longer with the oral route (43). Given the low success rate of intranasal midazolam alone, combining intranasal midazolam (0.2 mg/kg) with other agents, such as nitrous oxide–oxygen inhalation, has been recommended, achieving a satisfactory level of sedation in approximately two-thirds of pediatric dental cases in children aged 1.5–6 years (44).

Recent studies have compared nebulized inhaled midazolam with atomized intranasal midazolam. Children who received nebulized midazolam (0.5 mg/kg) via inhalation had greater sedation but a delayed onset compared with those who received atomized intranasal midazolam (0.3 mg/kg). The noninvasive nature of atomized intranasal midazolam may be more suitable for anxious children aged 3–5 years (5). Another study reported that nebulized buccal midazolam at 0.3 mg/kg and intranasal midazolam at 0.3 mg/kg were effective in managing uncooperative children aged 3 to 6 years during dental treatment, with a success rate of 85 and 80%, respectively (32). A burning sensation was reported in 9% of children with 0.3 or 0.5 mg/kg intranasal midazolam (28).

### Intranasal dexmedetomidine

In this review, the average success rate of intranasal dexmedetomidine in dental treatment was 75%. Intranasal dexmedetomidine (1–5 μg/kg) was associated with an average sedation onset time (21.10 min) and duration (70.36 min). The satisfactory sedation rate (80%) and behavior rate (79%) were high. In recent decades, intranasal dexmedetomidine has been used in premedication for general anesthesia and conscious sedation for pediatric procedures. Intranasal dexmedetomidine was primarily widely used as a premedication, and may be a safe and effective alternative in patients (mean age 5.5 years old) (45). Compared with oral midazolam, intranasal dexmedetomidine has better sedative effects of parent–child separation and anesthesia induction in pediatric premedication (46). Intranasal dexmedetomidine produces significant analgesia both during the intra- and post-operative period (27, 47). Intranasal dexmedetomidine appears to confer perioperative clinical sedation with improved postoperative analgesia for dental procedures under local anesthesia. In uncooperative children undergoing dental treatment, intranasal dexmedetomidine was associated with a significant reduction in pulse rate and systolic blood pressure compared with intranasal midazolam and ketamine, while all vital signs remained within physiological limits (27). Compared with intranasal midazolam (2.5 mg/kg), intranasal dexmedetomidine (1.5 μg/kg) demonstrated significantly better drug acceptance, a slower onset, and greater effectiveness for dental sedation in children aged 5–8 years (33).

### Oral sedation

The use of oral sedatives for pediatric dental treatment included midazolam (0.5–1 mg/kg), ketamine (4–5 mg/kg), and dexmedetomidine (4–5 μg/kg). Oral sedative was a slow-acting route and lasted 83.14 min. The satisfactory sedation rate ranged from 0 to 48.8%, and the average satisfactory sedation rate was 49%. The satisfactory behavior rate ranged from 18.2 to 83.3%, with an average rate of 45%. It was reported that a satisfactory level of sedation was achieved in approximately two-thirds of the pediatric dental cases using oral midazolam (0.5 mg/kg) combined with nitrous oxide-oxygen inhalation (44). Conscious sedation with midazolam (0.4 mg/kg or 0.5 mg/kg) and nitrous oxide is associated with an 80% sedation success rate (48). The oral route of drug administration is the most preferred route of drug administration as it is safe and convenient. Acceptance of the drug was better with oral rather than intranasal dexmedetomidine, although the onset of sedation was found to be better through intranasal than oral dexmedetomidine (35). The disadvantages of the oral route are slow onset of action, prolonged recovery, and high first-pass metabolism (43).

### Intranasal sedation combined with other sedatives

The intranasal sedatives include dexmedetomidine, sufentanyl, fentanyl, midazolam, ketamine, and esketamine. Intranasal esketamine or dexmedetomidine, combined with oral midazolam, was also described for pediatric dental sedation. The success rate of performing dental treatment (mean 84%), the satisfactory sedation rate (mean 83%), and the satisfactory behavior rate (mean 81%) were high. The combination of intranasal ketamine and midazolam improves the behavior of children compared with the same oral sedative combination as well as oral midazolam alone (37). In pediatric patients receiving intranasal dexmedetomidine combined with ketamine, ketamine (1 mg/kg) was administered 20 min after dexmedetomidine (2 μg/kg) to allow for the attainment of peak plasma concentrations of both drugs. Compared with intranasal dexmedetomidine (2.5 μg/kg) alone, the combination of dexmedetomidine and ketamine prolonged post-anesthetic recovery and reduced postoperative pain (36, 37).

Ketamine-containing intranasal regimens warrant particular emphasis—because ketamine provides dissociative analgesia and a rapid onset—complementing the anxiolysis of midazolam and the smooth sedation of dexmedetomidine. In terms of clinical efficacy, the intranasal midazolam–ketamine combination achieved a procedure success rate of 78.1% and a satisfactory sedation rate of 75.0%, while the intranasal dexmedetomidine–ketamine combination reached 93.8 and 100%, respectively (39); in an earlier triple-blind trial, intranasal ketamine alone provided a success rate of 66.7% and a satisfactory sedation rate of 76.2%, which was slightly higher than intranasal midazolam in the same study (27). For behavioral management, the intranasal ketamine–midazolam combination improved children’s behavior relative to the corresponding oral combination and to oral midazolam alone (37), and ketamine-containing combinations showed satisfactory behavior rates of 81–94% (39). With respect to recovery, adding ketamine to dexmedetomidine lengthened sedation duration (MD 26.0 min) and prolonged recovery (36), which may be advantageous for longer restorative procedures but less so for brief visits. Regarding safety, ketamine-containing regimens were generally well tolerated: Adverse events were predominantly mild nausea and vomiting, with desaturation in only 1.1% of children receiving dexmedetomidine alone or combined with ketamine (36), and no serious events were reported. These observations are reinforced by a recent crossover trial that could not be included in our quantitative synthesis: Dubey et al. (49) compared intranasal ketamine (7 mg/kg) with an intranasal midazolam (0.3 mg/kg)–dexmedetomidine (3 μg/kg) combination in 47 children aged 3–9 years and found that intranasal ketamine had a faster onset, a faster time to recovery, and higher patient acceptance, with comparable and acceptable physiological parameters and no postoperative complications. Taken together, ketamine-based intranasal regimens appear to be an effective and well-tolerated option for pediatric dental sedation. However, the optimal ketamine dose and combination partner remain to be defined.

Several clinically relevant crossover trials of intranasal sedation in pediatric dental patients were excluded from our quantitative synthesis (*n* = 16) because the carry-over of baseline anxiety and learned behavior between treatment visits would confound a within-subject comparison. We nonetheless acknowledge that these studies, including the trial by Dubey et al. (49) discussed above, contribute meaningful clinical evidence, particularly on ketamine-containing regimens, and their broad concordance with our pooled findings—favorable sedation and behavioral outcomes for intranasal routes with an acceptable safety profile—lends qualitative support to the present conclusions.

### Limitations

Several limitations should be acknowledged. First, head-to-head pairwise comparisons were based on a small number of trials per outcome (k = 1–4 across primary and secondary outcomes), with five outcomes contributed to by only a single trial; for these single-trial outcomes, the estimates should be interpreted descriptively rather than as meta-analytic findings. This scarcity of trials, together with the small sample sizes, limits the precision and the generalizability of the pooled estimates, and is reflected in the GRADE certainty (moderate to very low) reported in Supplementary Table S4. Second, the included studies were clinically and methodologically heterogeneous, differing in sedative agents, drug combinations, doses, delivery techniques (drops, atomizer, nebulizer), sedation and behavior scales, age range, and procedural setting; between-study heterogeneity was correspondingly substantial for several outcomes (I^2^ up to 70–100%). Although we used a random-effects model and harmonized the various sedation and behavior scales into common categories, this heterogeneity indicates that the pooled effects represent an average across diverse protocols rather than the effect of a single standardized regimen, and they should be extrapolated to any specific clinical setting only with caution; it also contributed to the downgrading for inconsistency in the GRADE assessment. Residual confounding cannot be excluded, and prespecified sensitivity analyses (leave-one-out, RCT-only, and risk-of-bias stratification) were not feasible because of the small number of trials per outcome. Third, single-arm summary rates per regimen are vulnerable to between-study confounding (for example, the apparently high oral-sedative rate observed across selected trials reflects a small subgroup of low-risk procedures and should not be read as evidence of superiority over intranasal routes); we, therefore, did not pool single-arm rates and report them only as study-level descriptive data within Tables 1, 2. Fourth, our search was restricted to PubMed, Web of Science and the Cochrane Central Register and did not cover Embase or non-English databases. Omitting Embase may have missed trials indexed there but not in the searched databases, and excluding non-English reports may have left out relevant regional pediatric sedation studies, particularly from settings where intranasal sedation is widely practiced; the resulting language and database bias is of undetermined magnitude. We restricted inclusion to English-language reports to ensure reliable data extraction and report this constraint transparently rather than leaving it implicit. Fifth, two non-randomized studies (21, 22) were appraised using ROBINS-I and judged at serious risk of bias in the confounding domain; their data were retained but should be interpreted with caution. Finally, the optimal dose, vehicle, and timing of intranasal sedatives in children remain to be defined; adequately powered head-to-head RCTs with standardized outcome definitions are needed.

## Conclusion

Intranasal sedatives, such as dexmedetomidine and midazolam, are clinically effective and well-tolerated alternatives to invasive sedation in children undergoing dental procedures. Head-to-head evidence shows that intranasal dexmedetomidine and intranasal midazolam achieve comparable procedure success and satisfactory sedation rates, although dexmedetomidine has a slower onset. Procedure success rates did not differ significantly between intranasal and oral regimens or between combined and single intranasal protocols. However, several between-comparison outcomes were informed by only one or two trials and should be regarded as preliminary. Combined sedative regimens may further improve sedation and behavioral outcomes; however, heterogeneity in drug, dose, and combination partner limits a firm recommendation. Adequately powered head-to-head randomized controlled trials with standardized outcome definitions are needed before a single optimal regimen can be endorsed for routine pediatric dental practice.

Future research on pediatric intranasal sedation should prioritize several directions. First, outcome reporting needs to be standardized: The field would benefit from a core outcome set with uniform definitions of procedure success, satisfactory sedation, and satisfactory behavior and from the consistent use of validated, comparable sedation and behavior scales, so that future trials can be pooled without the scale harmonization required in this study. Second, adequately powered, blinded head-to-head RCTs are required, particularly direct comparisons of ketamine-based regimens with dexmedetomidine- and midazolam-based regimens, which our review identified as a notable evidence gap. Third, dose-finding and dose–response studies are needed to define the optimal dose, vehicle, and timing for each agent and combination. Finally, future trials should report safety and recovery outcomes prospectively and in a harmonized manner, ideally registered in advance, to support higher-certainty evidence syntheses and trustworthy clinical recommendations.

## Data Availability

The original contributions presented in the study are included in the article/Supplementary material, further inquiries can be directed to the corresponding authors.
