# Supplementary material for: Intranasal dexmedetomidine versus midazolam for pediatric dental sedation: a pooled analysis of clinical trials
Source: Front Med (Lausanne). 2026 Jun 29;13:1882122. doi: 10.3389/fmed.2026.1882122 (PMC13358890; doi:10.3389/fmed.2026.1882122)
Supplement: Supplementary file 1 [file Table_1.DOCX]

| Supplementary Table S1. Detailed search strategies for each database | | | |
| --- | --- | --- | --- |
| Database | Search strategy | Search execution date | Number of hits |
| PubMed | (("Child"[MeSH Terms] OR ("paediatrics"[All Fields] OR "pediatrics"[MeSH Terms] OR "pediatrics"[All Fields] OR "paediatric"[All Fields] OR "pediatric"[All Fields]) OR ("Child"[MeSH Terms] OR "Child"[All Fields] OR "children"[All Fields] OR "child s"[All Fields] OR "children s"[All Fields] OR "childrens"[All Fields] OR "childs"[All Fields])) AND ("administration, intranasal"[MeSH Terms] OR ("intranasal"[All Fields] OR "intranasally"[All Fields])) AND ("Procedural Sedation"[MeSH Terms] OR ("sedate"[All Fields] OR "sedated"[All Fields] OR "sedating"[All Fields] OR "sedation"[All Fields] OR "sedations"[All Fields])) AND ("Dentistry"[MeSH Terms] OR ("dental health services"[MeSH Terms] OR ("dental"[All Fields] AND "health"[All Fields] AND "services"[All Fields]) OR "dental health services"[All Fields] OR "dental"[All Fields] OR "dentally"[All Fields] OR "dentals"[All Fields]) OR ("Dentistry"[MeSH Terms] OR "Dentistry"[All Fields] OR "dentistry s"[All Fields]))) AND (2014:2025[pdat]) | March 14, 2026 | 91 |
| Web of Science Core Collection | pediatric OR children (Topic) and intranasal (Topic)and sedation (Topic) and dentistry OR dental (Topic)and 2025 or 2024 or 2023 or 2022 or 2021 or 2020 or 2018 or 2019 or 2014 or 2015 or 2016 or 2017(Publication Years) | March 14, 2026 | 78 |
| Cochrane Central Register of Controlled Trials (CENTRAL) | (pediatric OR children):ti,ab,kw AND (intranasal):ti,ab,kw AND (sedation):ti,ab,kw AND (dentistry OR dental):ti,ab,kw (Word variations have been searched) with Publication Year from 2014 to 2025, with Cochrane Library publication date Between Jan 2014 and Dec 2025, in Trials | March 14, 2026 | 87 |
