# Supplementary material for: Intranasal dexmedetomidine versus midazolam for pediatric dental sedation: a pooled analysis of clinical trials
Source: Front Med (Lausanne). 2026 Jun 29;13:1882122. doi: 10.3389/fmed.2026.1882122 (PMC13358890; doi:10.3389/fmed.2026.1882122)
Supplement: Supplementary file 3 [file Table_3.DOCX]

| Supplementary Table S3. Excluded cross-over trials | | | | |
| --- | --- | --- | --- | --- |
|  | Author | Year of publication | Title | PMID |
| 1 | Sunbul N,  et al. | 2014 | Buccal versus intranasal midazolam sedation for pediatric dental patients | 25514077 |
| 2 | Musani IE,  et al. | 2015 | A comparison of the sedative effect of oral versus nasal midazolam combined with nitrous oxide in uncooperative children | 25939638 |
| 3 | Fallahinejad Ghajari M,  et al. | 2015 | Comparison of Oral and Intranasal Midazolam/Ketamine  Sedation in 3‒6-year-old Uncooperative Dental Patients | 26236429 |
| 4 | Mahdavi A,  et al. | 2018 | Intranasal Premedication Effect of Dexmedetomidine Versus Midazolam on the Behavior of 2-6-Year-Old Uncooperative Children in Dental Clinic | 29971125 |
| 5 | Srinivasan NK, et al. | 2021 | Comparison of the Sedative Effect of Inhaled Nitrous Oxide and Intranasal Midazolam in Behavior Management and Pain Perception of Pediatric Patients: A Split-mouth Randomized Controlled Clinical Trial | 35645472 |
| 6 | Musani I,  et al. | 2021 | Intranasal Midazolam Premedication for Anxiolysis in Children Reluctant to Receive Nitrous Oxide Sedation via Nasal Hood: An In Vivo Randomized Control Trial | 35645485 |
| 7 | Mowafy YN, et al. | 2021 | Efficacy of buccal versus intranasal route of administration of midazolam spray in behavior management of preschool dental patients | 34235908 |
| 8 | Shaat MA,  et al. | 2022 | Intranasal versus sublingual route of dexmedetomidine sedation in paediatric dentistry: A randomized controlled clinical trial | 34101918 |
| 9 | Alhaidari RI, et al. | 2022 | Intranasal Fentanyl Combined with Oral Midazolam for Pediatric Dental Sedation: A Controlled Randomized Blinded Crossover Clinical Trial | 34101918 |
| 10 | Alhaidari RI, et al. | 2022 | Post-Discharge Effects and Parents' Opinions of Intranasal Fentanyl with Oral Midazolam Sedation in Pediatric Dental Patients: A Cross-Sectional Study | 35204863 |
| 11 | Ann Preethy N, et al. | 2022 | Safety and physiologic effects of intranasal midazolam and nitrous oxide inhalation based sedation in children visiting Saveetha Dental College and Hospitals, India | 35815203 |
| 12 | Janiani P,  et al. | 2023 | Influence of Temperament on the Acceptance of Two Conscious Sedation Techniques in Toddlers Undergoing Dental Treatment: A Randomised Cross Over Trial | 37649959 |
| 13 | Janiani P,  et al. | 2023 | Assessment of Pain During Pediatric Dental Treatment Using Different Sedative Agents: A Crossover Trial | 37575859 |
| 14 | Ansari G,  et al. | 2024 | Evaluation of the sedative effect of intranasal versus intramuscular ketamine in 2-6-year-old uncooperative dental patients | 38375967 |
| 15 | Janiani P,  et al. | 2024 | Comparative evaluation of intranasal dexmedetomidine, intranasal midazolam, and nitrous oxide for conscious sedation of anxious children undergoing dental treatment: A randomized cross-over trial | 38957912 |
| 16 | Dubey B,  et al. | 2024 | Comparison of intranasal ketamine with intranasal midazolam and dexmedetomidine combination in pediatric dental patients for procedural sedation: A crossover study | 39250206 |
