# Supplementary material for: Intranasal dexmedetomidine versus midazolam for pediatric dental sedation: a pooled analysis of clinical trials
Source: Front Med (Lausanne). 2026 Jun 29;13:1882122. doi: 10.3389/fmed.2026.1882122 (PMC13358890; doi:10.3389/fmed.2026.1882122)
Supplement: Supplementary file 4 [file Table_4.DOCX]

**Supplementary Table S4. Summary of findings and GRADE certainty of evidence for the three head-to-head comparisons.**

**Comparison 1. Intranasal dexmedetomidine versus intranasal midazolam**

| **Outcome** | **Studies (participants)** | **Effect estimate (95% CI)** | **I²** | **Certainty (GRADE)** | **Reasons** |
| --- | --- | --- | --- | --- | --- |
| Procedure success rate | 3 (131) | RR 1.29 (0.86, 1.94) | 68% | **●●○○** Low | b, c |
| Onset time | 4 (207) | MD 8.79 (6.70, 10.87) min | 83% | **●●●○** Moderate | a |
| Sedation duration | 1 (63) | MD 20.32 (18.08, 22.56) min | NA | **●●●○** Moderate | c |
| Satisfactory sedation rate | 3 (131) | RR 1.04 (0.92, 1.16) | 35% | **●●●○** Moderate | c |
| Satisfactory behaviour rate | 2 (83) | RR 1.21 (0.94, 1.56) | 0% | **●●●○** Moderate | c |

**Comparison 2. Intranasal versus oral sedation regimens**

| **Outcome** | **Studies (participants)** | **Effect estimate (95% CI)** | **I²** | **Certainty (GRADE)** | **Reasons** |
| --- | --- | --- | --- | --- | --- |
| Procedure success rate | 2 (167) | RR 1.07 (0.99, 1.16) | 0% | **●●●○** Moderate | c |
| Onset time | 2 (127) | MD −15.17 (−49.26, 18.92) min | 97% | **●●○○** Low | b, c |
| Sedation duration | 1 (44) | MD 38.78 (11.74, 65.81) min | NA | **●○○○** Very low | a, d |
| Satisfactory sedation rate | 2 (127) | RR 4.41 (0.32, 60.04) | 73% | **●○○○** Very low | a, b, d |
| Satisfactory behaviour rate | 2 (128) | RR 1.97 (0.76, 5.14) | 76% | **●○○○** Very low | a, b, c |
| Adverse events | 2 (167) | RR 0.93 (0.51, 1.72) | 0% | **●●●○** Moderate | c |

**Comparison 3. Combined intranasal regimens versus single intranasal agents**

| **Outcome** | **Studies (participants)** | **Effect estimate (95% CI)** | **I²** | **Certainty (GRADE)** | **Reasons** |
| --- | --- | --- | --- | --- | --- |
| Procedure success rate | 2 (160) | RR 1.25 (0.73, 2.13) | 76% | **●●○○** Low | b, c |
| Onset time | 2 (160) | MD −3.68 (−8.72, 1.36) min | 84% | **●●○○** Low | b, c |
| Sedation duration | 1 (88) | MD 26.00 (17.32, 34.65) min | NA | **●●●○** Moderate | c |
| Satisfactory sedation rate | 2 (160) | RR 0.98 (0.91, 1.04) | 27% | **●●●○** Moderate | c |
| Satisfactory behaviour rate | 1 (88) | RR 1.08 (0.75, 1.56) | NA | **●○○○** Very low | a, d |
| Adverse events | 1 (88) | RR 1.29 (0.53, 3.15) | NA | **●●○○** Low | d |

**GRADE certainty of evidence: ●●●●** High **●●●○** Moderate **●●○○** Low **●○○○** Very low

CI, confidence interval; MD, mean difference; NA, not applicable (single study or I² not estimable); RR, risk ratio. All pooled estimates were derived from the randomised controlled trials; the two non-randomised studies contributed only to the single-arm descriptive data and not to these comparisons. Evidence from randomised trials started at high certainty and was rated down per outcome as indicated. No outcome reached high certainty because each estimate came from few small trials (optimal information size not met). Publication bias could not be assessed because every outcome was informed by fewer than ten studies.

**a.** Risk of bias — rated down one level because the trials contributing the majority of the weight for this outcome were at high or unclear risk of bias on a relevant domain (allocation concealment, or, for assessor-rated outcomes, blinding of outcome assessment). Outcomes whose contributing trials were at low risk on the relevant domains were not rated down for risk of bias.

**b.** Inconsistency — rated down one level for substantial unexplained statistical heterogeneity (I² ≥ 50% with inconsistent effect sizes). Onset-time outcomes were not rated down despite a high I² because every trial showed the same clinically important direction of effect.

**c.** Imprecision — rated down one level because the total sample size was below the optimal information size (including single-trial estimates) and/or the 95% confidence interval crossed the line of no effect.

**d.** Imprecision (very serious) — rated down two levels for an outcome informed by a single small trial with a wide or null-crossing interval, or, for satisfactory sedation in the intranasal versus oral comparison, an extremely wide interval (RR 4.41, 95% CI 0.32–60.04).
